# Easy and effective analytical method of carbendazim, dimethomorph, and fenoxanil from *Protaetia brevitarsis seulensis* using LC-MS/MS

**DOI:** 10.1371/journal.pone.0258266

**Published:** 2021-10-14

**Authors:** Sujin Baek, Hyun Ho Noh, Chang Jo Kim, Kyungae Son, Hee-Dong Lee, Leesun Kim

**Affiliations:** Residual Agrochemical Assessment Division, National Institute of Agricultural Sciences, Rural Development Administration, Wanju, Republic of Korea; Universita degli Studi del Molise, ITALY

## Abstract

Traditionally in Korea, *Protaetia brevitarsis seulensis* (white-spotted flower chafer) has been used as a medicine, and recently has attracted increased attention due to its antithrombotic efficacy. Some of spent mushroom compost or fermented oak sawdust, a feedstock for *P*. *brevitarsis*, were contaminated with three fungicides, carbendazim, dimethomorph, and fenoxanil, which could be transferred to the insect. This study was aimed to optimize a simple extraction method combined with liquid chromatography tandem mass spectrometry and apply it to the real samples. After the pulverized samples (5 g) were extracted with acetonitrile (10 mL) and formic acid (100 μL), fat and lipids in the samples were slowly precipitated at -20°C for 24 hours. After eight different clean-up methods were investigated, the mixture of 150 mg MgSO_4_/25 mg PSA/25 mg C18 was selected due to optimal recovery of the target compounds. Recovery (77.9%‒80.8% for carbendazim, 111.2%‒116.7% for dimethomorph, and 111.9%‒112.5% for fenoxanil) was achieved with reasonable relative standard deviation (<5.5%) The analytical method developed in this study was used to analyze three compounds in the 24 insect samples donated by the insect farm owners but no target compounds were detected. These results can provide important data for establishing the pesticide safety standards for *P*. *brevitarsis* before the medical applications.

## 1. Introduction

Edible insects have attracted attention as a future food source due to their high nutritional value of fat (11.88–25.14%) and proteins (44.23–58.32%) [1]. However, the priority issue of food safety of edible insects must be addressed such that the insects may be considered as a safe protein source or healthy supplements in future food industries. Possible contamination by pollutants and pesticide residues derived from their feed is a concern. Our previous studies optimized the analytical method for recovery of several pesticides from *Tenebrio molitor* larva (mealworms) [2, 3]. However, an analytical method has not been developed for pesticide analysis in *Protaetia brevitarsis seulensis* (white spotted flower chafer).

*P*. *brevitarsis* is one of several edible insects including beetles, grasshoppers, honeybees, mealworms, and silkworms that have received the Korean government support as a promising future protein source or for medicinal purposes [4]. In particular, *P*. *brevitarsis* has been used in Korean traditional medicines [5]. Recently, this insect has attracted more attention because it has been reported in several Asian countries that insect-derived protein hydrolysates have anticancer, antidiabetic, antiobesity, antioxidative, and hepatoprotective effects [6, 7]. It was also demonstrated to have antithrombotic efficacy [8] to prevent neurodegenerative disease [9]. Due to its medical effect, the fast analytical method of pesticide residue in *P*. *brevitarsis* is required in order to use the insect as nutritional supplements or medicine. It is challenging to extract pesticides from the flower chafer because *P*. *brevitarsis* contains fat (15–17%), protein (44%–58%) [1, 10], and various minerals including phosphorus, calcium, magnesium, sodium, and potassium [11], which also can be interference during instrumental analysis.

In combination with liquid chromatography tandem mass spectrometry (LC-MS/MS) and gas chromatography tandem mass spectrometry (GC-MS/MS), modified QuEChERS (quick, easy, cheap, effective, rugged, and safe) methods have been reported for extraction of pesticide multiresidues from different type of matrices [12–14]. Many studies used a single sorbent, sorbent mixture, and low-temperature precipitation to effectively eliminate fat and lipids from a wide range of fatty samples such as avocado [15], edible insects [2, 3], meat [16], honey bees [17], peanut oil [18], soybean-based products [19], and vegetable oils [20]. Our previous study used low temperature precipitation (-20°C) to remove large amount of fat and lipids derived from mealworms to analyze fenoxanil, deltamethrin, and chlorpyrifos [2]. In the clean-up procedure, C18 mixture with magnesium sulfate (MgSO_4_) and primary secondary amine (PSA) gave the best recoveries after various sorbents (C18, Zirconia based sorbent (Z-sep), Z-sep+, and EMR lipid) were investigated. However, some issues derived from high fat contents still remain due to the different behaviors of each pesticide in diverse samples [21]. Previous studies improved the recovery of lipophilic compounds by changing the ratio of solvent/sample to reduce matrix effects derived from a wide range of food matrices [20].

In this study, three fungicides, carbendazim, dimethomorph, and fenoxanil were selected as target compounds. After screening 320 pesticides in the feed made from spent mushroom compost or fermented oak sawdust for *P*. *brevitarsis*, three compounds were detected in the several feeds from the insect farms (not published yet). Of these fungicides, carbendazim has been applied as a broad-spectrum fungicide, to control fungal diseases in agriculture (e.g., cereals, grapes, lettuce, and pome and stone fruits), forestry, and veterinary medicines [22]. Dimethomorph is also widely used in more than 100 fruits and vegetables including wheat, onions, gingers, and cucumbers and fenoxanil is generally applied for rice in Korea based on the 2020 Pesticide Handbook [23]. However, carbendazim has been classified in the hazardous category of chemicals by the World Health Organization [22] and appears on the priority list of endocrine-disrupting chemicals from the European Commission [24]. Recently, it was reported that the nondegradable nature and extensive use of carbendazim have led to accumulation of carbendazim residues in the environment. In this regard, carbendazim monitoring in food commodities is urgently required [22, 25, 26]

This study was designed to optimize the analytical method of fenoxanil, dimethomorph, and carbendazim in *P*. *brevitarsis* using liquid chromatography tandem mass spectrometry (LC-MS/MS). Eight different clean-up procedures were investigated to remove the large amount of protein, fat, and minerals derived from the sample matrices. The optimized method was applied to the 24 real samples donated by the insect farms across the nation.

## 2. Materials and methods

### 2.1. Chemicals and solvents

Individual standards of carbendazim (98.5%), dimethomorph (98.1%), and fenoxanil (98.5%) were obtained from Dr. Ehrenstöfer (London, UK). High performance liquid chromatography (HPLC)-grade of methanol (MeOH), acetonitrile (MeCN), distilled water (DW), and formic acid (CH_2_O_2_) were purchased from Merck KGaA (Darmstadt, Germany). Extraction packets containing 4 g MgSO_4_, 1 g sodium chloride (NaCl), 1 g sodium citrate, 0.5 g disodium citrate sesquihydrate and dispersive solid phase extraction (d-SPE) containing 1) Fatty-EN (150 mg MgSO_4_/25 mg PSA/25 mg C18), 2) Fatty-AOAC (150 mg MgSO_4_/50 mg PSA/50 mg C18), 3) d-SPE-graphitized carbon black (GCB) (150 mg MgSO_4_/25 mg PSA/25 mg C18/25 mg GCB), mixture of 4) C18 20 mg/20 mg Z-sep, 5) 75 mg Z-sep alone, and 6) 75 mg Z-sep+ alone were obtained from Supelco (Bellefonte, PA, USA). Captiva EMR-lipid^TM^ (1 mL, 40 mg) cartridge was from Agilent Technologies (Santa Clara, CA, USA). OASIS and a PRiME hydrophilic-lipophilic balance (HLB) cartridge (1 cc, 30 mg) were from Waters (MA, USA). The contents of the two cartridges are not revealed by the company.

Spiking and working calibration solutions for LC-MS/MS analysis were prepared through dilution of the stock solutions with MeCN. All the prepared standard solutions were kept in a freezer (-20°C) for the analysis.

### 2.2. LC-MS/MS analysis

A liquid chromatograph (AB SCIEX Exion LC) equipped with tandem mass spectrometers MS/MS (AB SCIEX TQ5500) was operated for analysis of the target compounds. The analytes were separated on a non-polar column (Halo^®^ C18 2.1 mm i.d. × 100 mm L, 2.7 μm particle size, Waters, MA, USA) using mobile phase A (0.1% formic acid in DW) and mobile phase B (0.1% formic acid in MeOH). Injection volume was 1 μL. The column oven temperature was operated at 40°C. The first program started with 85% of mobile phase A for 18 min. The program for fenoxanil started with 75% of mobile phase B for 20 min. Detains of two different mobile phase gradient programs displayed in S1 Table were used for analysis of carbendazim/dimethomorph (S1A Table) and fenoxanil (S1B Table), respectively. The MS/MS was operated in negative electrospray ionization mode and multiple reaction monitoring conditions for LC-MS/MS, as listed at Table 1. The MS conditions were as follows: ionspray voltage of 5,500 V, nebulizer gas of 50 psi, curtain gas of 25 psi, drying gas of 50 psi, collision gas of 10 psi, and drying gas temperature of 500°C.

**Table 1:** Multiple reaction monitoring conditions of each target compound for LC-MS/MS analysis.

Target Compounds	Retention Time (min)	Qualitative Ion/ Confirmation Ion (*m/z*)	CE* (V)
Carbendazim	3.4	192.1>160.1	25
		192.1>132.0	41
Dimethomorph	7.1/7.6	388.0>301.0	24
		388.0>165.0	14
Fenoxanil	17.0	329.0>302.1	17
		329.0>86.0	31

*CE: collision energy.

### 2.3. Sample extraction and clean-up procedure

The sample extraction in this study was modified based on the QuEChERS method [12]. Five grams of sample was added in a 50-mL tube and 10 mL of DW was added to the samples for moisturizing. The samples were left for half an hour before adding 10 mL of MeCN. Then, samples were vigorously shaken by a Geno grinder (SPEX. Centiprep 1600 Mini G) for 1 min. The extraction packet was added to the sample and vigorously shaken by the grinder for 1 min. After the samples were centrifuged at 3,500 rpm for 15 min, they were stored at -20°C for 24 hours to effectively remove fat and lipid before further clean-up.

**Fig 1:**
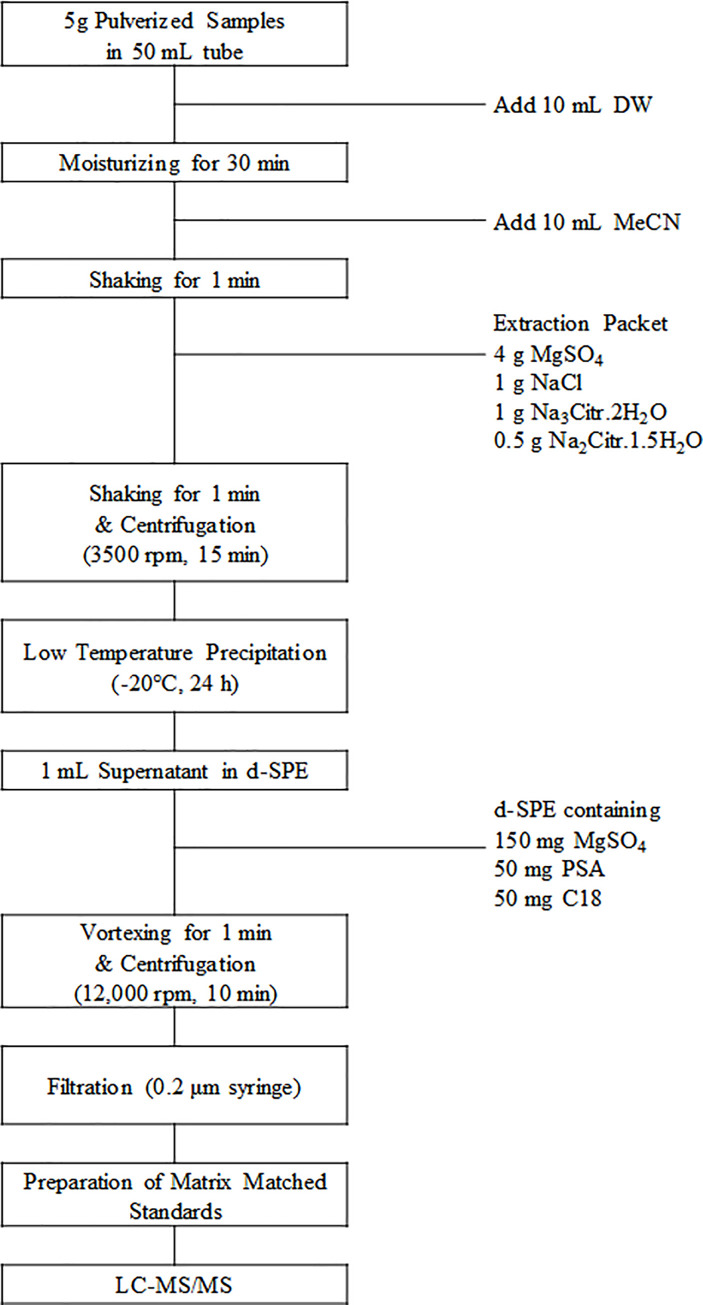
Sampling procedure optimized in this study.

Several different clean-up procedures were compared by obtaining recovery rates. Spiking level was at 0.05 mg/kg. The eight methods are described as follows: 1) d-SPE Fatty-EN; 2) d-SPE Fatty-AOAC; 3) d-SPE-GCB; 4) dSPE Z-sep/C18; 5) d-SPE Z-sep; 6) Z-sep+; 7) SPE EMR-lipid; and 8) SPE PRiME HLB. For clean-up, 1 mL of the extract upper layer was transferred into d-SPE (from 1 to 7 clean-up procedure) tube containing the mixture and shaken by vortexing for 1 min. Then, the samples were centrifuged at 12,000 rpm in 15 min. For SPE clean-up (7 and 8), 1 mL of the extract upper layer was transferred into a SPE cartridge and eluted into the 2-mL tube. All of the samples were filtered using a 0.22-μm syringe filter (PTFE filter membrane, Techno Plastic Products, Switzerland) before the analysis by LC-MS/MS. Fig 1 shows the final optimized method in this study.

### 2.4. Analytical method validation

The analytical method developed in this study was validated by obtaining mean recovery (%) and relative standard deviation (RSD, %) for evaluation of its accuracy, precision, repeatability, and reproducibility. All the experiments were carried out in triplicate at each spiking level. Repeatability and reproducibility were also confirmed by conducting recovery tests in another day (Intraday). At two spiking levels of 0.01 mg/kg and 0.05 mg/kg, recovery was obtained by plotting matrix-matched standards for LC-analysis. The fortification levels were decided due to the introduction of Positive List System (PLS) in Korea. Based on the PLS, in case that the pesticide in some food does not have maximum residue level (MRL) yet, they should follow one standard which is less than 0.01 mg/kg. Matrix-matched standards played an important role in minimizing the error occurring by instrumental signal enhancement or suppression by mixing blank matrix extracts with a solvent standard solution. For quantitative analysis of target compounds, the mixture of matrix-matched standards was prepared at the levels of 0.001, 0.0025, 0.005, 0.01, and 0.025 mg/kg. Linearity, limit of detection (LOD), and limit of quantitation (LOQ) were evaluated by plotting each matrix-matched calibration curve. LOD and LOQ were also calculated to be the lowest concentration having an S/N ratio with a peak of quantitative ion above 3 and 10 respectively. Finally, the 24 insect samples collected from the farms were analyzed using the method optimized in this experiment.

Matrix effect (ME) (%) was determined based on the following equation,

$$ME\;(\%)=(\frac{slope\;of\;matrix-matched\;standards\;curve}{slope\;of\;solvent\;standards\;curve}-1)\times 100$$

When the ME is around 0%, there is no matrix effect on the analytical results. When the ME is greater than 0%, there is ion suppression and when the ME is less than 0%, there is ion enhancement.

## 3. Results and discussion

### 3.1. Selection of a gradient program

**Fig 2:**
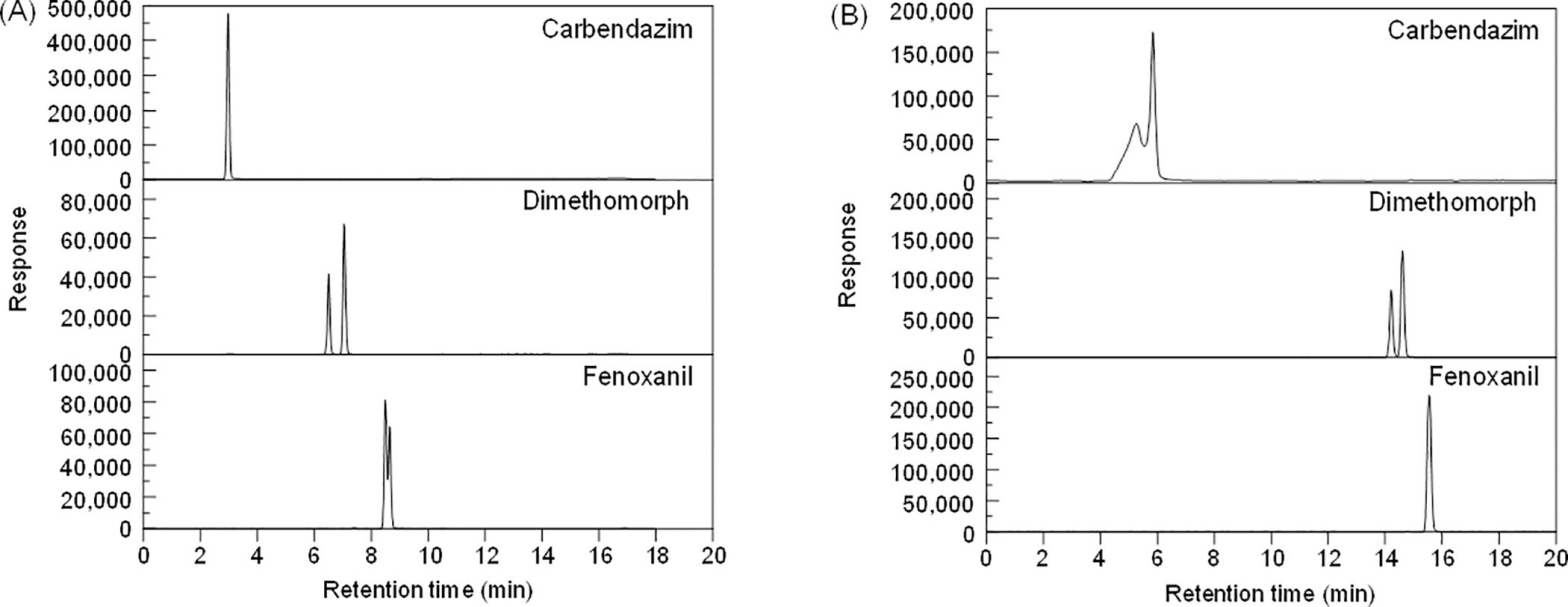
Chromatograms achieved with matrix-matched standards (0.01 mg/kg) of each compound after the clean-up procedure using a gradient program (A) generally used for multiresidue analysis, and (B) used for fenoxanil.

Simultaneous analysis of the three compounds using LC-MS/MS after using the same extraction method is ideal. Therefore, several gradient programs were investigated in this study, but it is challenging to optimize one gradient program for simultaneous analysis. Finally, different gradient programs with the same column and eluents were selected for carbendazim/dimethomorph (Fig 2A) and fenoxanil (Fig 2B). Using a multiresidue analysis program used in our group, carbendazim and dimethomorph showed a Gaussian peak shape and good sensitivity. Unlike the other two compounds, fenoxanil gave peak splitting, causing low response. Previous studies on multiresidue analysis generally quantified fenoxanil with a split peak even though it was less sensitive [27, 28]. However, with a different program (S1B Table), fenoxanil gave a Gaussian peak shape and better response while carbendazim showed peak fronting and splitting. In this program, the amount of organic eluent in the beginning of program was decreased from 85% to 75% and flow rate was also decreased from 0.3 mL/min into 0.1 mL/min.

### 3.2. Investigation of clean-up methods

**Fig 3:**
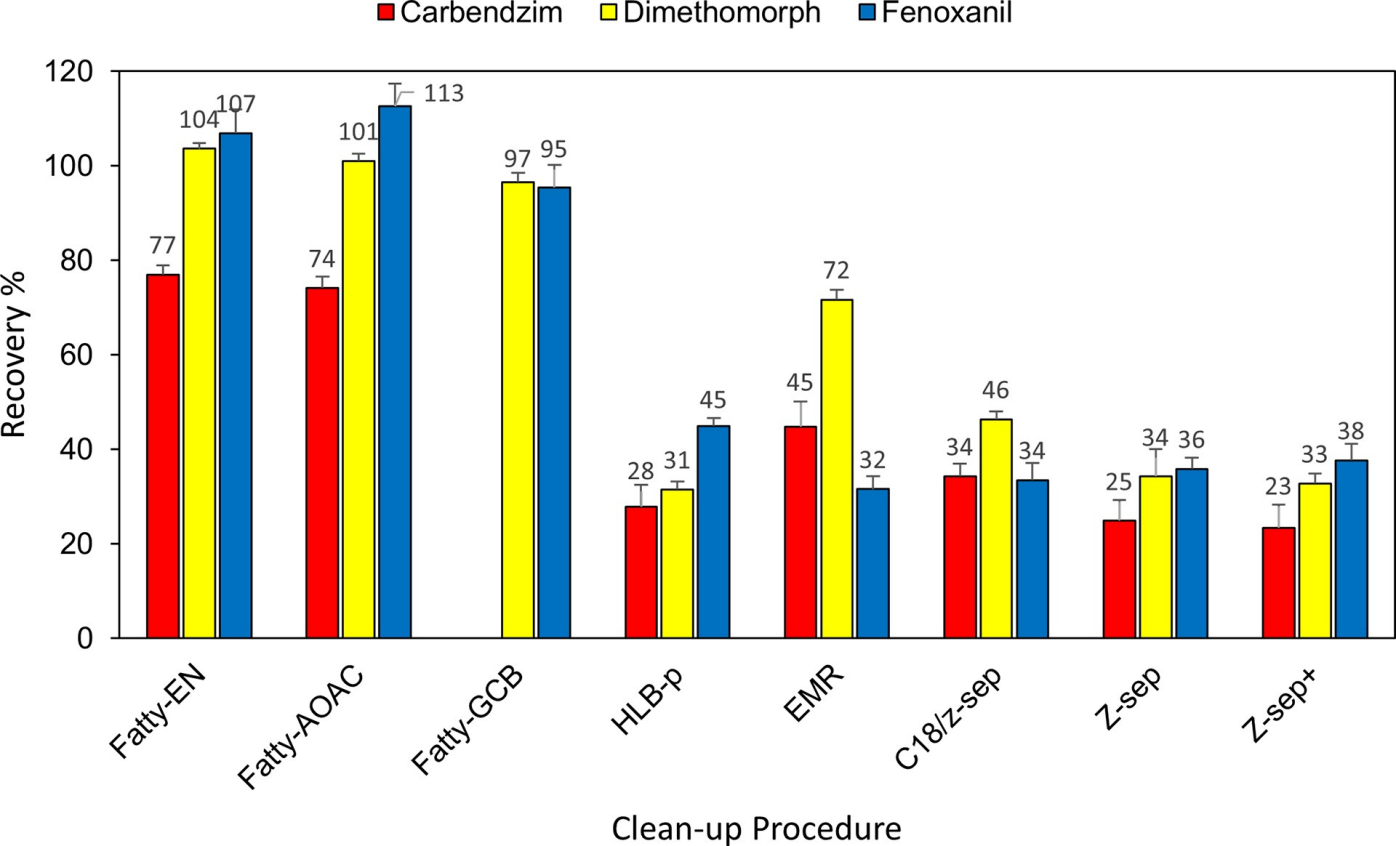
Recovery (n = 3) obtained after eight different clean-up procedures with the supernatants of acetonitrile extract. Spiking level was at 0.05 mg/kg.

Recovery of carbendazim, dimethomorph, and fenoxanil achieved after eight different clean-up procedures with the extract at the spiking level of 0.05 mg/kg is shown in Fig 3 and S2 Table. Protein and fat contents of edible insects proved the greatest interference challenge when pesticide residues are extracted from *P*. *brevitarsis*. This study investigated various clean-up methods, which have been applied for meal worms, because each pesticide responds differently when it is extracted from different samples. In this study, the mixture containing 150 mg MgSO_4_/25 mg PSA/25 mg C18 gave the best recovery for the target compounds, which is consistent with the previous study that successfully removed fat and lipids from meal worms [2]. For the analysis of tryphenlylene and chrysene in a wide range of foods such as those of animal origin with a moderate to high fat content, infant foods, and plant-derived products, the Z-Sep, mixture of MgSO_4_/PSA/C18, and EMR-lipid were compared but the mixture was selected for clean-up due to the cost-effectiveness and the availability of ready-to-use kits. Fatty-AOAC contained more PSA (25 mg more) and C18 (25 mg more) but recovery did not vary greatly from those of the Fatty-EN method. Several studies also investigated the efficiency of the EMR substances in pesticide analysis in terms of fat removal [21, 29].

Both SPE (PRiME HLB and EMR lipid) cartridges are suitable for removal of a large amount of fat, but the contents of both cartridges are proprietary. PRiME HLB has been reported to successfully eliminate various sources of interference including protein and fat for polar compounds such as glyphosate [30–32]. In this study, PRiME HLB gave low recoveries (27.9%‒44.9% ± 1.6%‒4.5%) for all of the target compounds. It was assumed that the material in the cartridge can retain the analytes of interest as well as the interference.

The three d-SPE (C18/Z-sep, Z-sep and Z-sep+) also investigated in this study (Fig 3) gave very low recoveries (33.5%‒34.4% ± 1.7%‒3.6% with C18/Z-sep, 25.0%‒35.9% ± 2.4%‒5.6% with Z-sep, and 23.4%‒37.7% ± 2.1%‒4.9% with Z-sep+). Z-sep is designed for removal of fat matrices and pigments and Z-sep+ is an advanced version of Z-sep but it was concluded that Z-sep and Z-sep+ absorbed the target compounds in this study.

GCB can effectively remove pigments (chlorophyll) [33]. A previous study showed that the addition of GCB to PSA and MgSO_4_ in the procedure of removal of chlorophyll from olive extracts increased from 64% of co-extractives (by weight) to 75% without compromise of recoveries [34]. However, it is not generally used in multiresidue analysis because it strongly adsorbs pesticides with planar ring structures such as carbendazim, chlorothalonil, thiabendazole, and hexachlorobenzene [35]. This study demonstrated that GCB completely retained carbendazim (no recovery obtained) but gave good recoveries for dimethomorph (96.6% ± RSD 1.9%) and fenoxanil (95.4% ± RSD 4.8%).

### 3.3. Matrix effect and method validation

**Fig 4:**
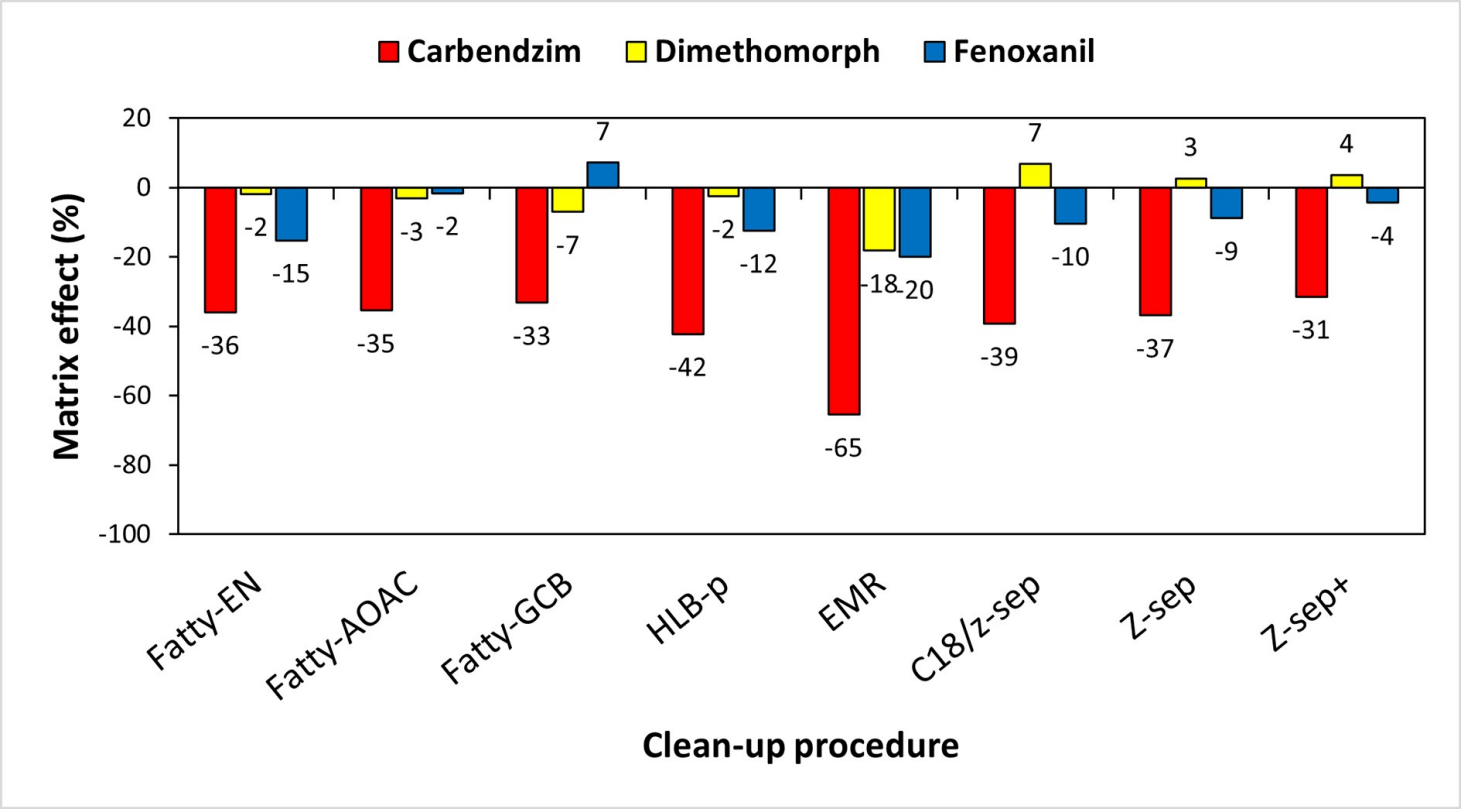
Matrix effects (%) were determined after analysis of carbendazim, dimethomorph, and fenoxanil in *P*. *brevitarsis* after eight different clean-up procedures with the sample acetonitrile extract.

MEs (%) are a major issue in quantitative analysis by LC-MS/MS because they can have a negative effect on accuracy, precision, and sensitivity of the analytical method applied. In this study, the MEs were determined for each target compound in *P*. *brevitarsis* after eight different clean-up procedures (Fig 4). The values of the matrix effect for all the target compounds are shown in Fig 4 and S2 Table. The MEs obtained in this study showed signal suppression, which is consistent with the fact that matrix co-eluting compounds in LC-MS/MS generally cause signal suppression while those in GC-MS/MS mainly lead to signal enhancement [36, 37]. As other studies have demonstrated [35, 38, 39], the MEs in this study were variable from compound to compound. Carbendazim gave the values from -65.4% to -31.4%. EMR Lipid clean-up gave the largest value, -65.4% for carbendazim. The mixture containing 150 mg MgSO_4_/25 mg PSA/25 mg C18, which was selected for clean-up in this study gave -35.9%. Comparing previous studies showing that ME of carbendazim was 30% in beewax [39] and 120% in rice [38], carbendazim in this experiment may not be greatly influenced by the sample derived matrices. MEs of dimethomorph were in the range from -18.1% to 6.8% (-1.8% from the Fatty-EN clean-up) which is insignificant, compared with the previous studies with over 100% in rice [38], brown rice, spinach, and orange with LOQ 1.0 ng/g [35]. MEs of fenoxanil are ranged from -19.9% to 7.2%, which is not significant. Previous study showed that MEs of fenoxanil in brown rice, spinach, orange, and potatoes were over 500% using modified QuEChERS combined with GC-MS/MS [35]. The optimized clean-up process gave ME of -15.2% for fenoxanil. It can be concluded that there was no significant interference from matrices, indicating that MEs did not cause peak identification or quantification issues. Plotting matrix-matched calibration curves played an important role in minimizing the matrix effect.

Recovery of carbendazim (77.0% ± RSD 2.0%) was lower than those of other compounds (106.9% ± RSD 5.0%) for fenoxanil, and 103.6% ± RSD 1.3% for dimethomorph; S2 Table). It may be due to higher ME of carbendazim. Previous study showed that recovery for carbendazim in beewax was 72% at the spiking level of 0.2 mg/kg and 78% at 0.1 mg/kg with 10 ng/g LOQ [39].

After optimization of the analytical method for this study, recovery studies were performed at two fortification levels of 0.01 mg/kg and 0.05 mg/kg for LC-MS/MS at replicates (n = 3) for two different days. Calibration curves presented linearity between 0.5–25 ng/mL for LC-MS/MS. All calibration curves displayed linearity with *r*^*2*^ > 0.99. All the pesticides studied gave recoveries (Table 2 and S3 Table) between 70% and 120% with the RSD lower than 20% for both fortification levels. In a different day, recovery at the two spiking levels (0.01 mg/kg and 0.05 mg/kg) for the target compounds was also achieved with 75% to 115% with the RSD less than 5.5% (S4 Table), demonstrating the intraday precision, reproducibility, and repeatability. The 24 real sample analyses using the developed method (S5 Table) showed the concentration of three compounds were lower than LODs, demonstrating that the target compounds detected in the feed were not transferred into the insect.

**Table 2:** Recovery of the target analytes obtained at the two spiking levels (0.01 mg/kg and 0.05 mg/kg).

Target Compounds	Spiking Level (mg/kg)	Recoveries (%)	RSD (%)	LOD (mg/kg)	LOQ (mg/kg)
Carbendazim	0.01	80.8	4.0	0.005	0.05
	0.05	77.9	0.8		
Dimethomorph	0.01	116.7	2.8	0.001	0.01
	0.05	111.2	3.0		
Fenoxanil	0.01	111.9	2.9	0.001	0.01
	0.05	112.5	5.2		

*RSD: relative standard deviation.

## 4. Conclusions

Recently, *P*. *brevitarsis* has attracted attention due to its medicinal properties; therefore, it is urgent to establish reliable analytical methods in order to monitor pesticide residues in this edible insect. This study described the optimization of a simplified and effective method for analysis of carbendazim, dimethomorph, and fenoxanil in *P*. *brevitarsis* using LC-MS/MS. The results showed that the target pesticides exhibited different responses to the clean-up adsorbents. The simple ready-to-use kit containing 150 mg MgSO_4_, 25 mg PSA, and 25 mg C18 was selected for clean-up after low precipitation due to the best recovery of the target. The analytical method optimized in this study can provide fast quantification for three fungicides on *P*. *brevitarsis* to provide a safe medicinal material for human consumption. Considering that the efficacy of *P*. *brevitarsis* has been demonstrated in many studies, this optimized analytical method will contribute to development of the edible insect industry, providing new economic opportunities to farmers in Korea.

## Supporting information

S1 Table A gradient program for (A) carbendazim/demethomorph and (B) fenoxanil.
(PDF)

S2 Table Recoveries, regression, and matrix effects of three target compounds obtained using 8 cleanup methods (at the spiking level of 0.05 mg/kg). (PDF)

S3 Table Final recoveries, regression, and matrix effect of three compounds at the two spiking level. (PDF)

S4 Table Final recoveries, regression, and matrix effect of three compounds at the two spiking level in a different day for intraday precision. (PDF)

S5 Table Concentration of three compounds in real samples after analyzing using the developed method. (PDF)
