# Supplementary material for: Easy and effective analytical method of carbendazim, dimethomorph, and fenoxanil from Protaetia brevitarsis seulensis using LC-MS/MS
Source: PLoS One. 2021 Oct 14;16(10):e0258266. doi: 10.1371/journal.pone.0258266 (PMC8516223; doi:10.1371/journal.pone.0258266)
Supplement: S1 Table — A gradient program for (A) carbendazim/demethomorph and (B) fenoxanil. (PDF) [file pone.0258266.s001.pdf]

S1 Table. A gradient program for (A) carbendazim/demethomorph and (B) fenoxanil

(A)

Time (min)	Initial	1.0	1.1	3.0	11.5	13.5	13.6	15.0	15.1	18.0
A (%)	85	85	40	40	10	10	2	2	85	85
B (%)	15	60	60	60	90	90	98	98	15	15

(B)

Time (min)	Initial	11	15	16	20
A (%)	75	10	10	75	75
B (%)	25	90	90	25	25
