# Supplementary material for: Easy and effective analytical method of carbendazim, dimethomorph, and fenoxanil from Protaetia brevitarsis seulensis using LC-MS/MS
Source: PLoS One. 2021 Oct 14;16(10):e0258266. doi: 10.1371/journal.pone.0258266 (PMC8516223; doi:10.1371/journal.pone.0258266)
Supplement: S2 Table — (PDF) [file pone.0258266.s002.pdf]

S2 Table. Recoveries, regression, and matrix effects of three target compounds obtained using 8 cleanup methods (at the spiking level of 0.05 mg/kg).

No	Target Compounds	Regression	ME*(%)	Recoveries (%)				
				1	2	3	Mean	RSD¥ (%)
dSPE Fatty-EN	Carbendazim	0.99997	-35.9	75.8	78.7	78.1	77.5	2.0
	Dimethomorph	0.99999	-1.8	103.6	104.2	102.4	103.4	0.9
	Fenoxanil	0.99982	-15.2	104.9	113.1	102.9	107	5.1
dSPE Fatty-AOAC	Carbendazim	0.99992	-35.4	72.8	75.6	76.4	74.9	2.5
	Dimethomorph	0.99999	-3.1	103.2	101.5	100.4	101.7	1.4
	Fenoxanil	0.99986	-1.5	108.5	118.7	110.6	112.6	4.8
dSPE Fatty-GCB	Carbendazim	0.99989	-33.2	No recovery				
	Dimethomorph	0.99999	-7.0	93.2	96.7	96.4	95.4	2.0
	Fenoxanil	0.99999	7.2	98.2	99.3	90.8	96.1	4.8
PRiME HLB	Carbendazim	0.99971	-42	28.8	26.5	28.5	27.9	4.5
	Dimethomorph	0.99857	-2	30.9	31.7	31.9	31.5	1.7
	Fenoxanil	0.99917	-12	44.7	45.7	44.3	44.9	1.6
EMR-lipid	Carbendazim	0.99564	-65.4	46.9	42.2	45.2	44.8	5.3
	Dimethomorph	0.97653	-18.1	73.2	70.3	71.6	71.7	2.0
	Fenoxanil	0.99792	-19.9	31	32.6	31.1	31.6	2.8
C18 20 mg/ Z-sep 20 mg	Carbendazim	0.98656	-39.2	35.1	33.3	34.4	34.3	2.6
	Dimethomorph	0.97856	6.8	47.2	46.5	45.7	46.5	1.6
	Fenoxanil	0.99934	-10.3	34.9	33.1	32.6	33.5	3.6
Z-sep 75 mg	Carbendazim	0.99928	-36.8	24.5	24.3	26.3	25.0	4.4
	Dimethomorph	0.99966	2.7	35.4	32.1	35.6	34.4	5.7
	Fenoxanil	0.99839	-8.8	36.9	35.4	35.3	35.9	2.5
Z-sep+ 75 mg	Carbendazim	0.99611	-31.4	24.1	22.1	24.1	23.4	4.9
	Dimethomorph	0.99606	3.7	26.6	25.4	25.2	25.7	2.9
	Fenoxanil	0.99742	-4.4	37.9	38.9	36.3	37.7	3.5

1. dSPE (Fatty-EN): PSA 25 mg, C18 25 mg, $MgSO_4$ 150 mg
2. dSPE (Fatty-AOAC): PSA 50 mg, C18 50 mg, $MgSO_4$ 150 mg
3. dSPE (Fatty-GCB): PSA 25 mg, C18 25mg, GCB 50 mg, $MgSO_4$ 150 mg

*ME: matrix effect; ¥RSD: relative standard deviation;
