# Supplementary material for: Easy and effective analytical method of carbendazim, dimethomorph, and fenoxanil from Protaetia brevitarsis seulensis using LC-MS/MS
Source: PLoS One. 2021 Oct 14;16(10):e0258266. doi: 10.1371/journal.pone.0258266 (PMC8516223; doi:10.1371/journal.pone.0258266)
Supplement: S3 Table — (PDF) [file pone.0258266.s003.pdf]

S3 Table. Final recoveries, regression, and matrix effect of three compounds at the two spiking level.

Spiking level (ng/g)	Target Compounds	Regression	ME*(%)	Recoveries (%)				
				1	2	3	mean	RSD¥ (%)
10	Carbendazim	0.99998	-43.2	77.5	84.0	80.8	80.8	4.0
	Dimethomorph	0.99999	-1.2	115.4	120.4	114.3	116.7	2.8
	Fenoxanil	0.99960	-5.8	109.9	115.7	110.2	111.9	2.9
50	Carbendazim	0.99998	-43.2	77.7	77.4	78.6	77.9	0.8
	Dimethomorph	0.99999	-1.2	113.8	107.5	112.3	111.2	3.0
	Fenoxanil	0.99960	-5.8	107.0	112.0	118.5	112.5	5.2

*ME: matrix effect; ¥RSD: relative standard deviation;
