# Supplementary material for: Easy and effective analytical method of carbendazim, dimethomorph, and fenoxanil from Protaetia brevitarsis seulensis using LC-MS/MS
Source: PLoS One. 2021 Oct 14;16(10):e0258266. doi: 10.1371/journal.pone.0258266 (PMC8516223; doi:10.1371/journal.pone.0258266)
Supplement: S4 Table — (PDF) [file pone.0258266.s004.pdf]

S4 Table. Final recoveries, regression, and matrix effect of three compounds at the two spiking level in a different day for intraday precision.

Spiking level (ng/g)	Target Compounds	Regression	ME*(%)	Recoveries (%)				
				1	2	3	mean	RSD¥ (%)
10	Carbendazim	0.99998	-45.2	81.1	70.0	79.4	76.8	7.8
	Dimethomorph	0.99303	-1.5	83.5	78.5	76.7	79.6	4.5
	Fenoxanil	0.99536	-6.1	111.6	104.9	112.9	109.8	3.9
50	Carbendazim	0.99998	-45.2	75.2	77.1	74.9	75.7	1.6
	Dimethomorph	0.99303	-1.5	102.8	105.3	105.1	104.4	1.3
	Fenoxanil	0.99536	-6.1	109.7	116.9	108.2	111.6	4.2

*ME: matrix effect; ¥RSD: relative standard deviation;
