# Supplementary material for: Easy and effective analytical method of carbendazim, dimethomorph, and fenoxanil from Protaetia brevitarsis seulensis using LC-MS/MS
Source: PLoS One. 2021 Oct 14;16(10):e0258266. doi: 10.1371/journal.pone.0258266 (PMC8516223; doi:10.1371/journal.pone.0258266)
Supplement: S5 Table — (PDF) [file pone.0258266.s005.pdf]

S5 Table. Concentration of three compounds in real samples after analyzing using the developed method.

Feed	No	Concentration (mg/kg)		
		Fenoxanil	Carbendazim	Dimethomorph
Mushroom compost	1	< 0.01	< 0.01	< 0.01
	2	< 0.01	< 0.01	< 0.01
	3	< 0.01	< 0.01	< 0.01
	4	< 0.01	< 0.01	< 0.01
	5	< 0.01	< 0.01	< 0.01
	6	< 0.01	< 0.01	< 0.01
	7	< 0.01	< 0.01	< 0.01
	8	< 0.01	< 0.01	< 0.01
	9	< 0.01	< 0.01	< 0.01
	10	< 0.01	< 0.01	< 0.01
	11	< 0.01	< 0.01	< 0.01
	12	< 0.01	< 0.01	< 0.01
	14	< 0.01	< 0.01	< 0.01
	15	< 0.01	< 0.01	< 0.01
	16	< 0.01	< 0.01	< 0.01
	17	< 0.01	< 0.01	< 0.01
	18	< 0.01	< 0.01	< 0.01
	19	< 0.01	< 0.01	< 0.01
Fermented oak tree sawdust	1	< 0.01	< 0.01	< 0.01
	2	< 0.01	< 0.01	< 0.01
	3	< 0.01	< 0.01	< 0.01
	4	< 0.01	< 0.01	< 0.01
	5	< 0.01	< 0.01	< 0.01
